# Electrospun network based on polyacrylonitrile-polyphenyl/titanium oxide nanofibers for high-performance supercapacitor device

**DOI:** 10.1038/s41598-024-56545-w

**Published:** 2024-03-20

**Authors:** El-Refaie Kenawy, Youssef I. Moharram, Fatma S. Abouharga, Mona Elfiky

**Affiliations:** 1 Polymer Research Group, Department of Chemistry, Faculty of Science, Tanta University, Tanta, 31527 Egypt; 2 Analytical and Electrochemistry Research UNIT, Department of Chemistry, Faculty of Science, Tanta University, Tanta, Egypt

**Keywords:** Chemistry, Energy science and technology

## Abstract

Nanofibers and mat-like polyacrylonitrile-polyphenyl/titanium oxide (PAN-Pph./TiO_2_) with proper electrochemical properties were fabricated via a single-step electrospinning technique for supercapacitor application. Scanning electron microscopy (SEM), scanning transmission electron microscopy (STEM), thermogravimetry (TGA), fourier transform infrared (FTIR), X-ray diffraction (XRD) and energy dispersive X-ray (EDX) were conducted to characterize the morphological and chemical composition of all fabricated nanofibers. Furthermore, the electrochemical activity of the fabricated nanofibers for energy storage applications (supercapacitor) was probed by cyclic voltammetry (CV), charge–discharge (CD), and electrochemical impedance spectroscopy (EIS). The PAN-PPh./TiO_2_ nanofiber electrode revealed a proper specific capacitance of 484 F g^−1^ at a current density of 11.0 A g^–1^ compared with PAN (198 F g^−1^), and PAN-PPh. (352 F g^−1^) nanofibers using the charge–discharge technique. Furthermore, the PAN-PPh./TiO_2_ nanofiber electrode displayed a proper energy density of 16.8 Wh kg^−1^ at a power density (P) of 2749.1 Wkg^−1^. Moreover, the PAN-PPh./TiO_2_ nanofiber electrode has a low electrical resistance of 23.72 Ω, and outstanding cycling stability of 79.38% capacitance retention after 3000 cycles.

## Introduction

Supercapacitors have been among the best power sources for wearable devices because of their quick charge–discharge rates, exceptional power density, and reversible electrochemical properties. However, some supercapacitors have a low energy density, which limits their use. Therefore, the development of energy storage technology is essential to obtaining high efficiency, a long cycle life, low cost, and pollution-free energy. Recently, high-performance supercapacitors modified with diverse structures of nanomaterials and nanoparticles semiconductors have been developed and used as secondary batteries, owing to their high-power density and long lifetime compared to rechargeable batteries^1^. In particular, previously published reports have demonstrated that TiO_2_ nanoparticles (NPs) acts as an electron channel when combined with conductive poly aniline nanofibers (NFs), which improve electron and ion transit during the charge–discharge process^2^. Furthermore, TiO_2_ NPs have the ability to enhance the cyclic stability of electrodes required to improve the action of supercapacitors^3,4^.

Furthermore, conductive polymer nanocomposites, a carbon-based material, transition metal oxides, and polymer nanofibers (NFs) with high specific capacitance have recently been used to obtain high-performance electrode materials for supercapacitors^5^. Particularly, conductive polymers (conductive macromolecules) are being developed as a new kind of electrode material due to their high electrical conductivity, minimal internal resistance, and high specific capacity^6^. Whereas the main benefit of using conductive polymer materials in supercapacitors is their high working voltage (3.0 to 3.2 V). Some recent research innovations have focused on the improvement of the capacitance efficiency of the supercapacitor electrodes with nanocomposites based on TiO_2_ NPs dispersed in conductive polymers such as polypyrrole (PPy), polyacenes (PAS), polyaniline (PANI), polyacrylonitrile (PAN), and polythiophene (PTH)^5,7–12^. For instance, Bal Sydulu Singu et al. have successfully created a novel supercapacitor with a specific capacitance (C_sp_) value of 525 F g^−1^ based on the combination of multi-walled carbon nanotubes (MWNTs) and titanium oxide (TiO_2_) with polyaniline (PANI) (PANI–MWNTs–TiO_2_) to improve the specific capacitance performance and cycle stability of PANI (210 F g^−1^), and MWNTs (30 F g^−1^) electrodes^6^. Noteworthy, PANI-MWNTs-TiO_2_ demonstrated a huge surface area, owing to their morphological structures^6,13^, which contributes to the supercapacitor's good energy density.

On the other hand, the electrospinning process is considered to be one of the most recent simple and inexpensive methods for the production of nanofibers (NFs) accompanied by a high specific surface area^14^ from different morphological nanostructured materials such as organic polymers (e.g. polyvinylidene fluoride (PVDF), polymethacrylate (PMA)^15^, polyacrylonitrile (PAN)^12^, polystyrene (PS)^16^, and polyvinyl alcohol (PVA)^14,15^, inorganic metal oxides (e.g. TiO_2_, CuO, NiO, Fe_2_O_3_, LiMn_2_O_4_ and NiFe_2_O_4_), carbon materials (e.g., graphite, graphene and carbon nanotube (CNT)^17^, and organic/inorganic composites^18^ (e.g., carbon/SnO_2_, PVA/TiO_2_, collagen/hydroxyapatite, nylon-6/gelatin and graphene/TiO_2_)^19^. Noteworthy, the morphologies of electrospun NFs are strongly affected by the processing parameters chosen in electrospinning, solution^14^, and environmental parameters^16,20^. To obtain bead-free and symmetric fibers, controlling the type and properties of the injected solution, including the viscosity, concentration, and conductivity, is very important^21,22^.

Since polyacrylonitrile (PAN) can provide dimensionally stable film formation, its supercapacitor and conductivity performance are practically nil. But other proper features, including low cost, mechanical stability, and ability to form doped polymer make it suitable for use in high-efficiency supercapacitors^23^. For instance, in the Abdah, M., etc. report, the diameter of electrospun polyacrylonitrile (PAN) fibers presented a rather stable linear relationship with the applied voltage in the range of 10–20 kV^13^. Generally, the applied voltage increases with increasing molecular weight, owing to the increasing viscosity of the solution^24^. Moreover, the electrospun NF is also affected by a variety of important environmental parameters^25^. Notably, the shape and degree of porosity of electrospun fibers of different polymers are influenced by the humidity in the spinning environment^26^. High levels of humidity can cause jet surface charge leakage, impairing the tensile process and causing the fiber diameter to increase. Furthermore, the solvent on the tip will evaporate too quickly if the humidity level is too low, causing tip blockage^27^. Electrospun conductive polymer and semi-crystalline thermoplastic polymer electrode materials provide larger current densities as a benefit over carbon-based materials^28,29^.

To date, there has been no detailed research work on the fabrication of co-mixed polyacrylonitrile (PAN) and polyphenyl (PPh.) nanofibers. While Sema Aslan and et al.^5^ successfully fabricated a novel supercapacitor via electrospinning of PAN nanofiber in the presence of TiO_2_ NPs upon the surface of a discharged battery coal (DBC) electrode. The developed electrode achieved a C_sp_ value of 156.00 F g^−1^, which is comparatively high compared to other reported studies that have been published without the presence of TiO_2_ NPs. Furthermore, the creation of PAN nanofiber onto the surfaces of pencil graphite (PGE) and DBC electrodes has been successfully fabricated^5^. Noteworthy, the PAN nanofiber-coated DBC achieved a greater Csp value than the PAN/PGE electrode (74.93 F g^−1^).

In this context, polyacrylonitrile-polyphenyl/titanium oxide (PAN-PPh./TiO_2_) NF was synthesized for the first time via a single-step electrospinning technique to form nanofiber with unique electrochemical features, which could be suitable in the application of supercapacitors. Whereas a GCE modified using prepared nanofibers was successfully synthesized followed by the study of capacitive efficiency performance.

## Experimental

### Materials

Reagent-grade chemicals were purchased in their pure form and used as received: titanium (IV) oxide (TiO_2_, 99.0%, with particle size = 10μm), polyacrylonitrile (PAN, MW = 150.000), N,N–dimethylformamide (DMF, 99.0%), benzene (C_6_H_6_, 99.0%), aluminum chloride (AlCl_3_, 98.0%), cupric chloride (CuCl_2,_ 99.0%), hydrochloric acid (HCl, 37.0%), sodium hydroxide (NaOH, 99.0%), polymeric perfluorosulfonic acid (nafion, (5.0%), and potassium hydroxide (KOH, 98.0%).

### Instruments

(Burker, TENSOR 27-series FTIR, Germany) were used to obtain fourier transform infrared (FT-IR) spectra of all the fabricated NFs samples in the range of 400–4000 cm^−1^. Thermal stability, maximum degradation temperature, and change in mass with an increase in temperature were all measured via the thermal analyzer Perkin Elmer 4000 with a heating rate of 10.0 deg/min in the range of 50–800 °C. Cyclic voltammetry (CV), Electrochemical impedance spectroscopy (EIS), and charge–discharge measurements were carried out using a computer-controlled potentiostat/galvanostatic model CS3104 (China) in the microanalysis unit at Faculty of Science, Tanta university. Furthermore, XRD patterns of all the fabricated samples were measured in Tanta University's central laboratory in Egypt via an XRD instrument (300 Unisantis, Germany) conducted with Cu-K radiation (λ ≈ 1.5406, scanning rate of 0.05/sec at 45 kV and 0.8 mA). The morphological and chemical composition of fabricated samples were investigated using SEM, EDX instruments (JEOL Japan, JSM 6510LV) and STEM (Quattro S, ThermoFisher, USA).

### Synthesis of titanium (IV) oxide nanoparticles

The proper amount of purchased TiO_2_ particles was milled using the ball milling technique for 12 h. Subsequently, the resulting fine powder was collected, and the particle size was estimated via SEM.

### Synthesis of polyphenyl

P-polyphenyl was synthesized from benzene-aluminum chloride-cupric chloride with high care to prevent contamination. In brief, the reaction was conducted in a 3-necked flask with a paddle stirrer under N_2_. A mixture of (1: 0.5: 0.5 mol)/ 1 mL of (benzene: AlCl_3_: CuCl_2_) was mixed and injected into a 3-necked flask. The temperature was raised to 37 °C, and the reaction was continuously stirred for 30 min in the presence of an acidic gas. Followed by the addition of deionized water (DW), and then the reaction mixture was filtered. The product was first treated with an 18.0% diluted HCl solution, subsequently boiled with a concentrated HCl solution, and rinsed with DW until colorless. Afterword, the resulting polymer was treated twice through boiling with a 2.0 M NaOH solution, rinsed with DW until colorless, and negative response with a chloride ion. The resulting polymer appeared in the form of a finely split, light brown powder after drying at 120 °C for 5 h^30^.

### Preparation of the polyacrylonitrile nanofiber

A 10.0 wt.% PAN solution was prepared as follows: 1.0 g of PAN was dissolved in 10 mL of DMF with steady stirring for 2 h at 80 °C to disrupt the strong intra- and interchain bonding that may exist in the PAN polymer. Then, the polymer solution with a viscosity value of 8703 CP (8.703 P_a_ s) was inserted into a 10-mL plastic syringe equipped with a 0.4-mm needle diameter. The syringe tip was connected to a positive electrode (anode), and a negative electrode (cathode) was attached to a metallic collector covered by aluminum foil. The distance between the syringe tip and the collector was fixed to be 15 cm, and the applied potential was fixed at 14 kV.

### Preparation of the polyacrylonitrile and polyphenyl nanofiber

A 2.08 wt.% PPh., and 8.33 wt% of PAN solution were prepared as follows: 0.25 g of PPh. was dispersed in 12 mL of DMF by sonication for 1 h, and then 1 g of PAN was added to the sonicated solution with continuous stirring for 2 h at 80 °C. The prepared composite solution with a viscosity value of 2838 CP (2.838 P_a_ s) was then inserted into a 10-mL plastic syringe equipped with a 0.4-mm needle diameter. The syringe tip was connected to a positive electrode (anode), and a negative electrode (cathode) was attached to a metallic collector covered by aluminum foil. The distance between the syringe tip, and the collector was fixed to be 15 cm, and the applied potential was fixed at 14 kV.

### Preparation of nanofiber composite by electrospinning technique

4.17 wt% TiO_2_, 2.08 wt% of PPh. and 8.33 wt% PAN solutions were prepared as follows: 0.5 g of TiO_2_ NPs and 0.25 g of PPh. were dispersed in 12 mL of DMF by sonication for 1 h, and then 1 g of PAN was added to the sonicated solution with continuous stirring for 2 h at 80 °C. After that, the prepared composite solution with a viscosity value of 2691 CP (2.691 P_a_ s) was loaded into a 10-mL plastic syringe equipped with a 0.4-mm needle diameter. A high voltage of 14 kV, with a tip-collector distance of 15 cm, was applied to the solution and fiber collected on the metallic collector.

### Electrochemical measurements

A GCE with a 3.0 mm diameter was polished with 0.05 µm alumina powder to obtain a mirror-shiny surface and rinsed well before use. Then, 0.1 mg of PAN-PPh./TiO_2_ nanofiber was added to the surface of GCE, followed by the addition of a 5 µL mixture of [1.0 (nafion): 1.0 (isopropyl alcohol) mL] upon the surface of the sensor and the resultant [PAN-PPh./TiO_2_] nanofibers GCE were dried at 60 °C for 2 h. The same procedure was carried out to obtain [PAN] and [PAN-PPh.] nanofibers GCEs. The electrochemical measurements were carried out in 10.0 mL of 1.0 M KOH using a three-electrode system including Hg/Hg_2_Cl_2_ and platinum electrodes as reference and counter electrodes, respectively. CV measurements were estimated in the range of -1.50 V to 0.50 V using a 50 mVs^-1^ scan rate, and the EIS measurements were carried out in the range of 0.1 to 10^6^ Hz. Furthermore, specific capacitance (C_s_) values were evaluated for [PAN], [PAN-PPh.], and [PAN-PPh./TiO_2_] nanofibers GCEs using the following Eqs. (1) and (2):

1 $${C}_{s}=\frac{area\, under\, curve}{m\, \Delta V v}$$

2 $${C}_{s}=\frac{I\, \Delta T}{\Delta V}.$$

In which, *v*/ m V s^−1^ was a scan rate, ΔV/V, m/mg, I/A, and ΔT/s displayed the range of applied potential, mass of sample upon the surface of the sensor, current value of charge discharge, and time of charge discharge, respectively.

Moreover, the energy density and power density of [PAN], [PAN-PPh.], and [PAN-PPh./TiO_2_] nanofibers GCEs were evaluated according to the following equations:

3 $$E=\frac{C({\Delta V)}^{2}}{2*3.6}$$

4 $$P=\frac{3600E}{\Delta t}.$$

In which, *E*/ Wh kg^−1^ and P/W kg^−1^, was an energy density, and power density, respectively.

## Results and discussion

### Characterization of prepared nanofibers

#### FT-IR and XRD analysis of nanofibers

**Figure 1:**
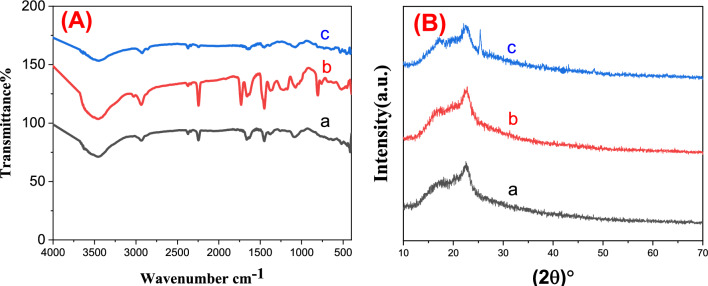
(**A**) FT-IR spectra and (**B**) XRD patterns of (a) PAN, (b) PAN-PPh. and (c) PAN-PPh./TiO_2_ nanofibers.

The FTIR spectra of PAN, PAN-PPh., and PAN-PPh./TiO_2_ nanofibers were obtained to provide more details about the formed electrospun materials^31^, as displayed in (Fig. 1A). The spectra show characteristic broad bands in the range of (3640–2500 cm^−1^), and bands at (2933.2, and 2870 cm^−1^), which are assigned to stretching *v*_OH_, and asymmetric and symmetric *v*_C–H_ in CH, CH_2_, and CH_3_ groups in all prepared nanofibers^32^, respectively. As shown in (Fig. 1Aa), the FTIR spectrum of PAN nanofiber displayed absorption bands at 2245, 1450.21, 1378.85, 1240, 1088.62 cm^−1^ can be assigned to stretching *v*_C≡N_, (*v*_CH3_, and scissor *v*_CH2_), symmetric *v*_CH3_ in C–CH_3,_ stretching *v*_C–N_, and bending *v*_C–N_. Furthermore, absorption bands at 1648, 1590, 1488 and 1450 cm^−1^ can be attributed to aromatic stretching *v*_C–C_ while a strong band at 1220 cm^−1^ owing to stretching of Ph-O-Ph in the aromatic ether chains^32^, as demonstrated in (Fig. 1Ab). Noteworthy, the FTIR spectrum of PAN-PPh./TiO_2_ nanofiber (Fig. 1Ac) displayed a little shift in the last mentioned bands, owing to the interaction between the contents of NFs. Moreover, according to a number of studies^6,33^, the FTIR spectrum of TiO_2_ displayed a band at 510 cm^−1^, corresponds to Ti–O.

Furthermore, the crystallinity phase of PAN, PAN-PPh., and PAN-PPh./TiO_2_ nanofibers was investigated using the XRD pattern, as presented in (Fig. 1B). In (Fig. S2), the XRD pattern of PPh. displayed well-defined diffraction bands at 2θ ≈ 19.9°, 22.78°, 28.0°, and 43.0°. As shown in (Fig. 1Ba), the XRD pattern of PAN nanofiber^33^ exhibited the presence of broad bands at 16.9° and 22.5°. Moreover, the PAN-PPh./TiO_2_ nanofiber pattern displayed a new sharp band at 2θ ≈ 25.36°, which can be related to TiO_2_ NPs.

#### Investigation of morphological structure

**Figure 2:**
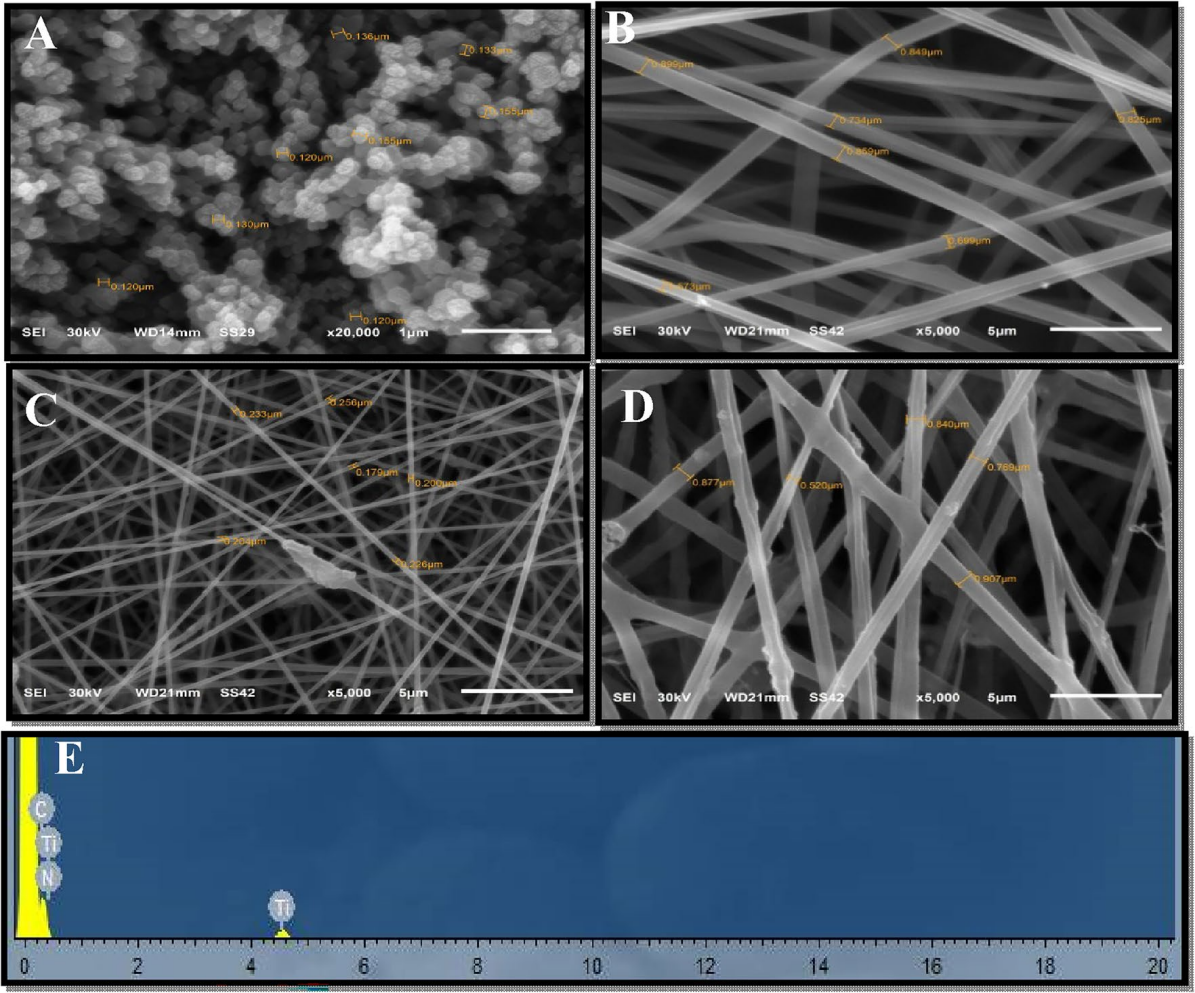
SEM images of (**A**) TiO_2_ NPs, (**B**) PAN nanofiber, (**C**) PAN-PPh. nanofiber, (**D**) PAN-PPh./TiO_2_ nanofiber, and (**E**) EDX spectrum of PAN-PPh./TiO_2_ nanofiber.

The SEM micrograph of TiO_2_ NPs in (Fig. 2A), clarified spherical particles with an average size of 162 nm. The NF average diameter is 921.0233, 217.9857, and 832.5509 nm for PAN, PAN-PPh., and PAN-PPh./TiO_2_ nanofibers, respectively. The EDX spectrum dispersion results showed oxide and titanium to be present in fiber (Fig. 2) the peak indicating the presence of carbon in the sample. The percentages of the elements carbon, nitrogen, oxygen, and titanium in the samples are 43.60, 49.10, 6.11, and 1.20%, respectively. The X-ray diffraction analysis (XRD) was done to ensure the accuracy of the results.

**Figure 3:**
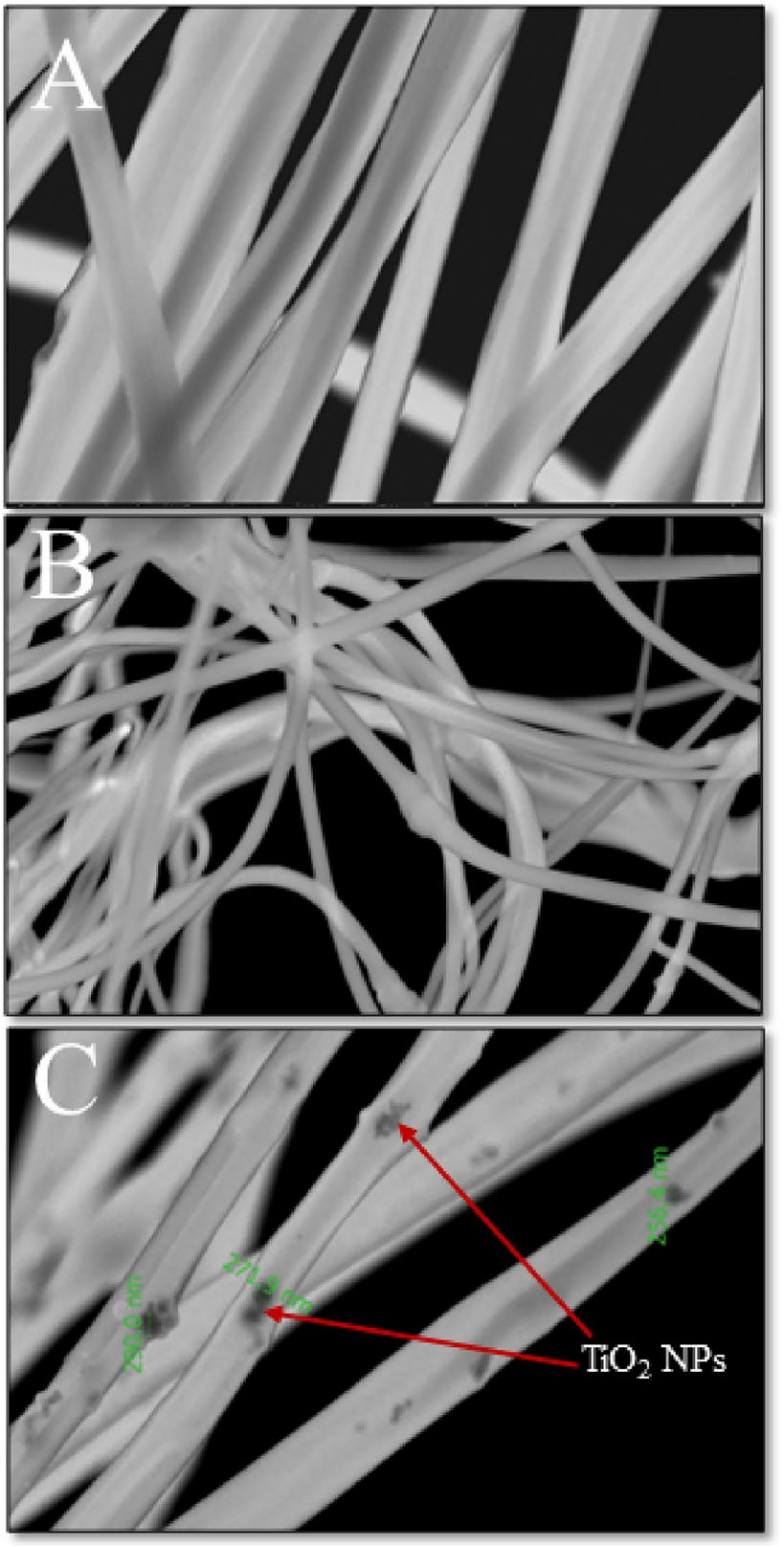
STEM micrographs of (**A**) PAN, (**B**) PAN-PPh. and (**C**) PAN-PPh./TiO_2_ nanofibers.

The STEM analysis of PAN, PAN-PPh., and PAN-PPh./TiO_2_ nanofibers was carried out to obtain more information about the morphological structure, as demonstrated in (Fig. 3). As shown in (Fig. 3A,B), micrographs of PAN, and PAN-PPh. showed that the diameters of the nanofibers were not uniform. Moreover, the PAN-PPh./TiO_2_ nanofiber micrograph (Fig. 3C) displayed a uniform nanofiber composed of irregular aggregates of TiO_2_ nanoparticles with an average size of 239.4 nm.

#### Thermal gravimetric analysis of nanofibers

**Figure 4:**
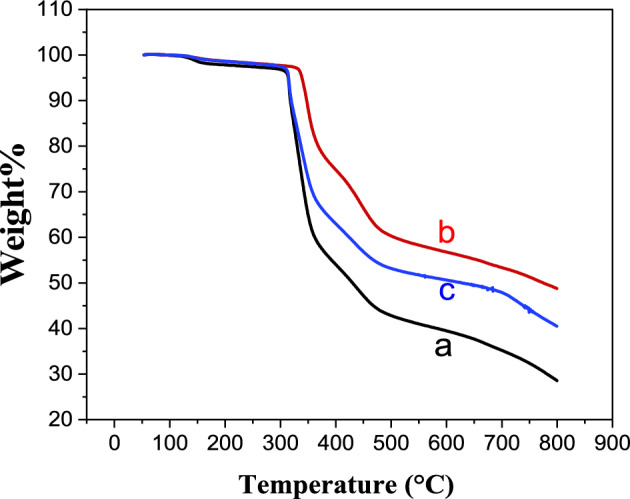
TGA curves of (**a**) PAN, (**b**) PAN-PPh. and (**c**) PAN-PPh./TiO_2_ nanofibers.

TGA analysis of PAN, PPh., PAN-PPh., and PAN-PPh./TiO_2_ nanofibers were carried out under N_2_ gas to estimate their degree of thermal resistance, as displayed in (Fig. 4 and Fig. S1). As shown in (Fig. 4), all prepared NFs were decomposed into the steps of composition observed in the range of 50 and 800 °C with total mass losses of 70.8, 48.24, and 59.7% for PAN, PAN-PPh. and PAN-PPh./TiO_2_ nanofibers, respectively. The resultant thermal analysis curve of PPh. has the lowest degree of thermal analysis compared with other NFs. Moreover, the thermal decomposition of PAN-PPh. nanofiber was delayed compared with PAN nanofiber, owing to its thermal stability, which rises with the degree of branching in the polymer, and can be increased further by a preparatory heat treatment in the absence of air^34^. While PAN-PPh./TiO_2_ nanofiber has moderate thermal stability due to the presence of TiO_2_ NPs accompanied by the PAN and PPh. nanofiber structures, promoting thermal stability, is necessary for the development of supercapacitors^5^. In the first/second regions of thermal decomposition, the weight loss of NFs reached 3.44/36.74, 2.5/18.58, and 2.54/31.77% for PAN, PAN-PPh. and PAN-PPh./TiO_2_ nanofibers, respectively. At the third/ fourth regions of thermal decomposition, the weight loss of NFs reached 16.43/14.49, 16.38/10.78, and 12.26/13.13% for PAN, PAN-PPh. and PAN-PPh./TiO_2_ nanofibers, respectively.

### Electrochemical characterization

Recently, the electrochemical performance of SCs devices has been improved by the development of new materials in a specific electrolyte. Therefore, cyclic voltammetry (CV), galvanostatic charging/discharging (GCD), and electrochemical impedance spectroscopy (EIS) have been probed to explore the specific capacitance, degree of resistance, and outstanding stability of fabricated electrodes. To explore the supercapacitive behavior of the fabricated NFs, cyclic voltammograms of [PAN], [PAN-PPh.], and [PAN-PPh./TiO_2_] nanofibers GCEs (Fig. 4A) were recorded in 10.0 mL of 1.0 M KOH at a *v* of 50 mV.s^−1^ accompanied by the use of Hg/Hg_2_Cl_2_ and platinum electrodes as reference and counter electrodes, respectively.

**Figure 5:**
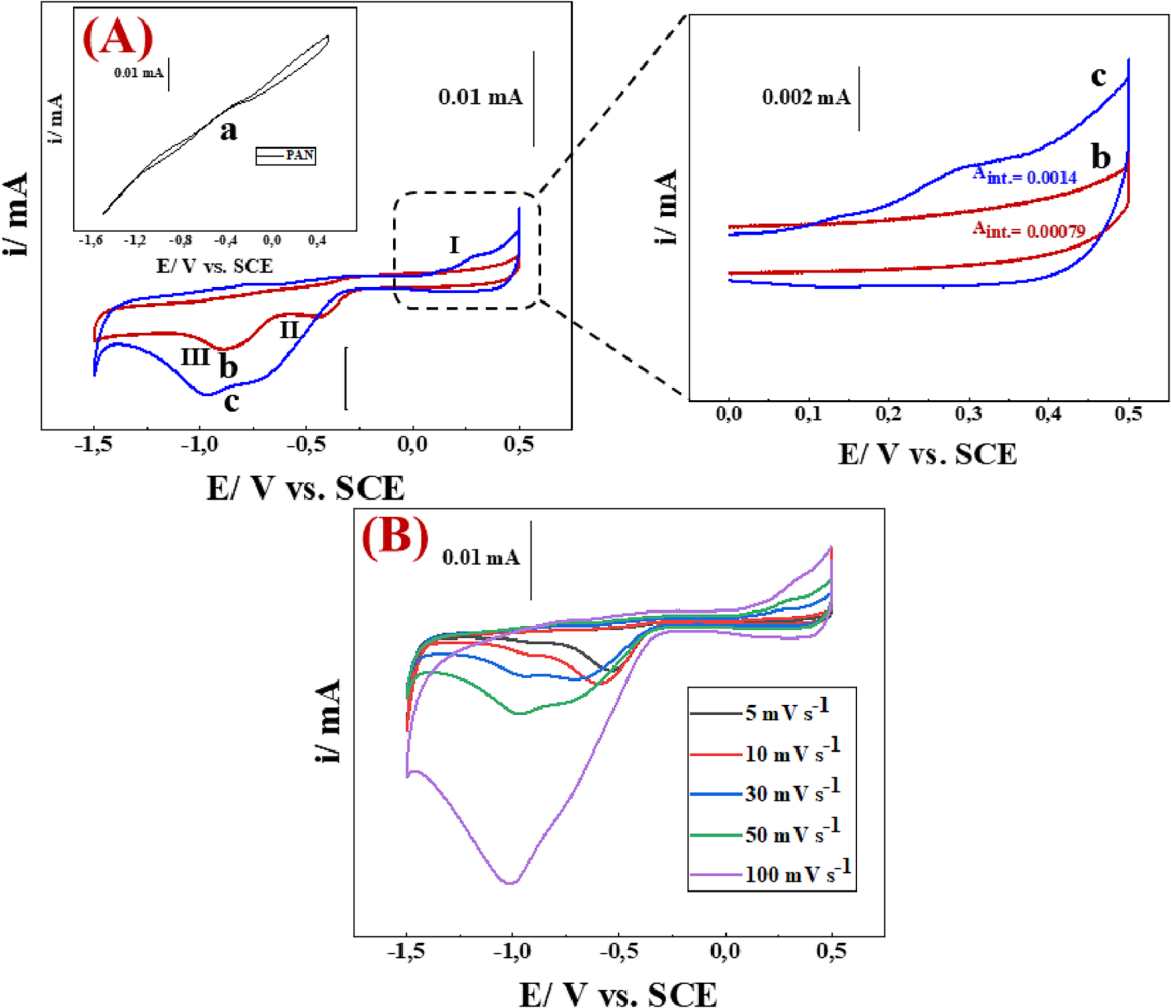
(**A**) cyclic voltammetry (CV) of (a) [PAN], (b) [PAN-PPh.], and (c) [PAN-PPh./TiO_2_] nanofibers GCEs at scan rate (***v***) of 50 mV s^−1^, (**B**) PAN-PPh./TiO_2_ composite electrode at various scan rate values (*v* mV s^−1^).

As shown in (Fig. 5A), the cyclic voltammogram of [PAN] has a very low capacitance current without the presence of any redox peaks. Otherwise, cyclic voltammograms of [PAN-PPh.], and [PAN-PPh./TiO_2_] nanofibers GCEs (Fig. 5A) display outstanding electrochemical performance and good capacitance characteristics, owing to the presence of [PPh.] and TiO_2_ compounds, which resulted from the conductive properties of PPh. It was postulated that the positive charge produced after doping delocalizes along the backbone, and this hole was discovered to form on top of the valence band. The formation of charges causes local disruption around the flaws. This process results in the creation of a localized band gap between the HOMO and the LUMO of conductive polymers (CPs) as a result of the LUMO downshift and the HOMO upshift. PPh. contains a conjugated orbital overlap structure, which results in continuous electron transport along the polymer backbone's main chain. If charge carriers are present, this conjugated molecule structure allows for effective charge transmission throughout the chain^35^. TiO_2_ is an n-type semiconductor that is employed in a variety of applications. TiO_2_ an amorphous crystalline, tetragonal rutile, and anatase TiO_2_ structure that possesses a low charge transfer resistance and a high specific capacitance^36^. The C_sp_ values of [PAN], [PAN-PPh.], and [PAN-PPh./TiO_2_] nanofibers GCEs are 220, 315.2, and 525 F g^−1^ at a current density of 11.0 A g^–1^, respectively. Noteworthy is the presence of voltmmteric peaks (P_II_, _III_), and (P_I_, _II_, _III_) in [PAN-PPh.], and [PAN-PPh./TiO_2_] nanofibers GCEs signify a higher capacity that could arise mainly from the aqueous electrochemical (Ox./Red.) processes of the phenolic group of PPh.^37^, and TiO_2_, respectively. These performances are strongly affected by the electrolyte energy levels, and the overlap of the electronic levels on the composite surface^38^. The [PAN-PPh./TiO_2_] nanofiber GCE exhibits a substantially larger capacitive current density than the [PAN], [PAN-PPh.] nanofibers GCEs, resulting in superior electrochemical capacitive performance. Furthermore, one of the most necessary requirements to evaluate the capacitive behavior of the proposed electrode is exploring the voltammteric response at various *v* (mV s^−1^) values. As displayed in (Fig. 5B), the cyclic voltammograms of [PAN-PPh./TiO_2_] nanofiber GCE were recorded at various scan rates (5, 10, 30, 50, and 100 mV s^−1^). At *v* of 100 mV s^−1^, the voltammogram clearly exhibited an increase in the current intensity of P_III_ accompanied by the disappearance of P_II_, and a decrease in the current value of P_I_, proving fast electron transfer upon the surface of the proposed electrode.

**Figure 6:**
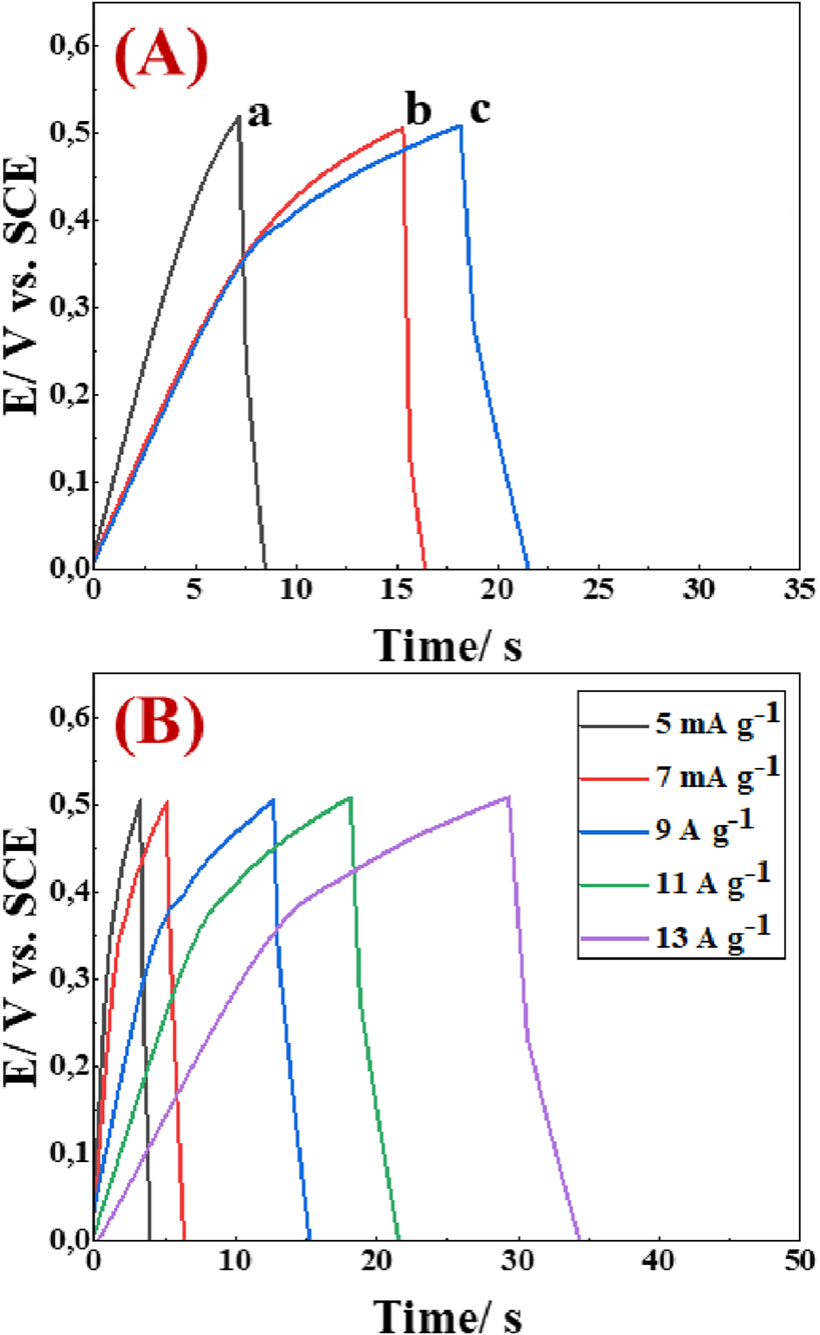
(**A**) GCD curves of (a) [PAN], (b) [PAN-PPh.], (c) [PAN-PPh./TiO_2_] nanofibers GCEs, and (**B**) [PAN-PPh./TiO_2_] nanofiber GCE at different a current density values (A g^−1^).

To further investigate the capacitive behavior of the fabricated electrodes, galvanostatic charge–discharge measurements were carried out^39^, as depicted in (Fig. 6). As displayed in (Fig. 6A), the charge–discharge curves of [PAN], [PAN-PPh.], and [PAN-PPh./TiO_2_] nanofibers GCEs were recorded in a 1.0 M KOH electrolyte at a current density of 11 A g^−1^. The C_sp_ values of [PAN], [PAN-PPh.], and [PAN-PPh./TiO_2_] nanofibers GCEs are 198, 352, and 484 F g^−1^, respectively, which is in good agreement with CV results. Furthermore, the specific capacitance of [PAN-PPh./TiO_2_] nanofiber GCE at different current densities ranging from 5.0 to 100 mV s^−1^ was recorded. From the GCD curves, [PAN-PPh./TiO_2_] nanofiber GCE reveals proper C_sp_ values of 40 to 910 F g^−1^ at 5.0 and 100 mV s^−1^, respectively.

Furthermore, the PAN-PPh./TiO_2_ nanofiber GCE displayed the highest energy density (E) value of (16.8 Wh kg^−1^) at a power density (P) of 2749.1 W kg^−1^ compared with [PAN] (6.875 Wh kg^−1^), and [PAN-PPh.] (12.2 Wh kg^−1^) nanofibers GCEs.

**Figure 7:**
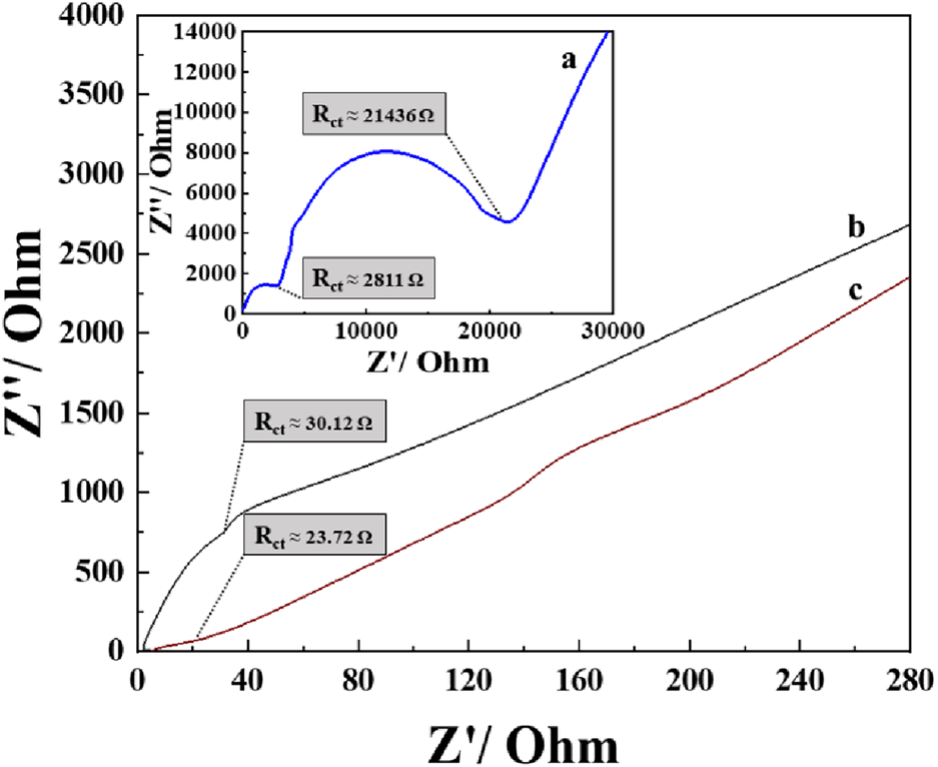
Nyquist plots of (**a**) [PAN], (**b**) [PAN-PPh.], and (**c**) [PAN-PPh./TiO_2_] nanofiber GCEs.

Since the capacitive efficiency of the proposed electrode is mainly dependent on the degree of resistance (R_ct_). The electrochemical impedance spectroscopy (EIS) and corresponding Nyquist plots have been recorded in a frequency range of 0.1 to 10^6^ Hz to evaluate the R_ct_ of the fabricated electrodes, as displayed in (Fig. 7). Whereas the semicircles in the Nyquist plots represent the charge transfer resistance (R_ct_)^4^, which is related to their surface characteristics. As shown in (Fig. 7), the R_ct_ values of EIS measurements are found to be 2811, 30.12, and 23.72 Ω for [PAN], [PAN-PPh.], and [PAN-PPh./TiO_2_] nanofibers GCEs, respectively.

**Figure 8:**
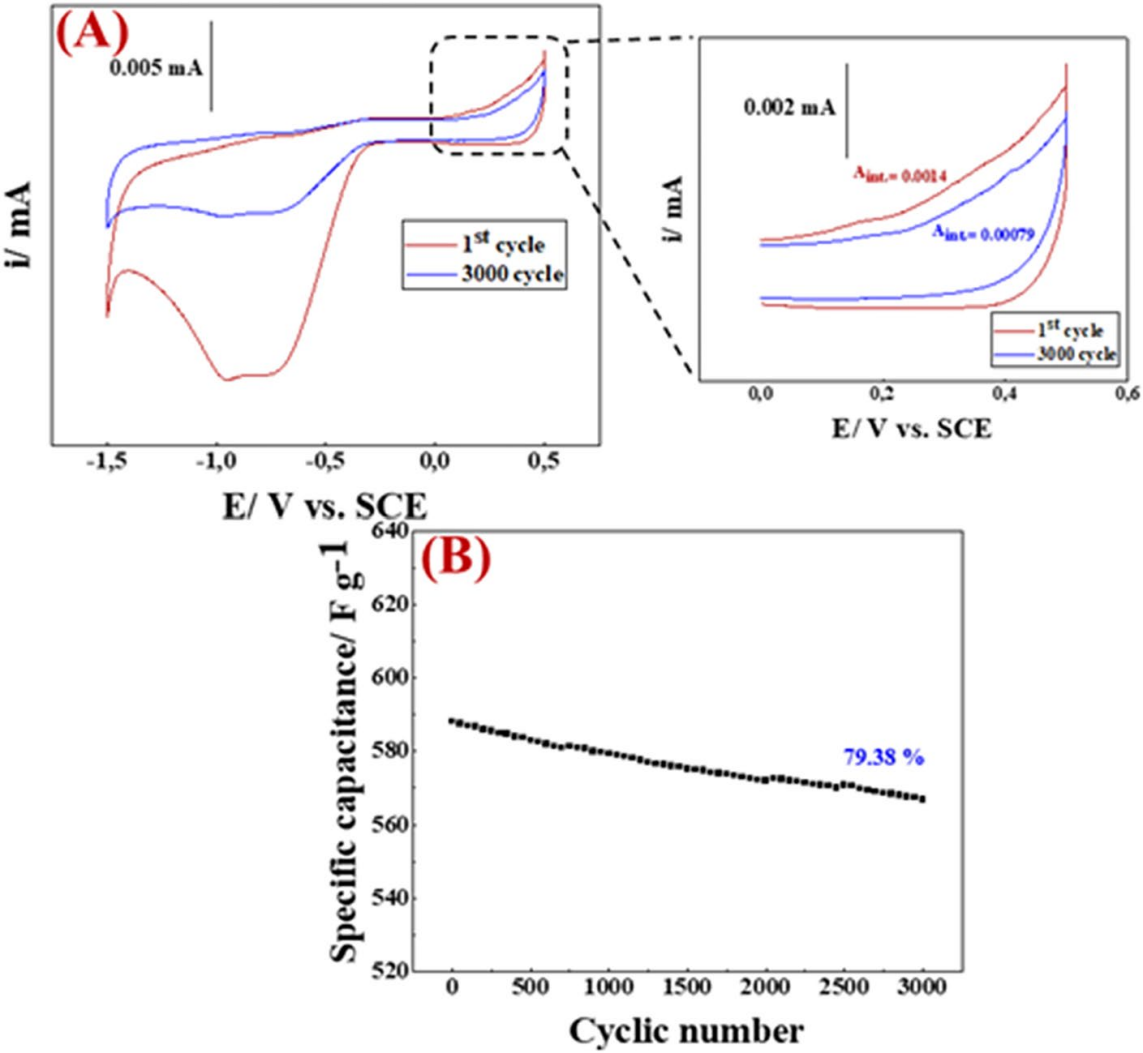
The cyclic stability of PAN-PPh./TiO_2_ electrode for 3000 cycles.

Moreover, the cycle life test (stability) of [PAN-PPh./TiO_2_] nanofiber GCE was obtained for 3000 cycles at *v* = 100 mV s^−1^ and a current density of 11.0 A g^–1^, as depicted in (Fig. 8). As shown in (Fig. 8A), the 1st cycle and after 3000 cycles recorded C_sp_ values of 525, and 416 F g^−1^, respectively. According to the last mentioned results, [PAN-PPh./TiO_2_] nanofiber GCE exhibits the greatest cycling performance with 79.38% capacitance retention after 3000 cycles, as plotted in (Fig. 8B). When comparing PAN-PPh./TiO_2_ with other nanocomposite or other compounds that contain TiO_2_ in the matrix structure (Table 1), we observed that the synthesized PAN-PPh./TiO_2_ nanofiber has impressive electric properties among all the previously reported nanocomposites.

**Table 1:** Comparison of the cyclic stability of TiO_2_ and their composite.

Compounds	Cyclic stability (%)	Refs
[TiO_2_ NFs] CF	94	^40^
PVP-TiO_2_ NFs	78	^41^
MoS_2_@TiO_2_	98	^42^
TiO_2_-MnO_2_ MP	61.3	^43^
(TiO_2_@MnO_2_) NS	81	^44^
V_2_O_5_/ TiO_2_	92	^45^
PANI/nTiO_2_/AC	72	^46^
PANi/Mn-TiO_2_	91	^47^
TiO_2_/CDC composite	85.1	^48^
PAN-Pph./TiO_2_	79.38	This study

Titanium dioxide nanoflowers ([TiO_2_-NFs]), carbon fabric (CF), Molybdenum disulfide (MoS_2_), Manganese oxide (MnO_2_), TiO_2_ NPs-embedded mesoporous MnO_2_ (TiO_2_-MnO_2_ MP), Titanium dioxide@manganese dioxide nanosheets (TiO_2_@MnO_2_ NS), Vanadium pentoxide (V_2_O_5_)/titanium dioxide (TiO_2_), Polyaniline/nano titanium dioxide/activated carbon (PANI/nTiO_2_/AC), Polyaniline-wrapped, manganese-doped titanium oxide (PANi/Mn-TiO_2_), Spherical titanium oxide/carbide-derived carbon (TiO_2_/CDC) composite, polyacrylonitrile-polyphenyl/titanium oxide (PAN-PPh./TiO_2_).

According to all the preceding characteristics of fabricated electrodes, we can conclude that [PAN-PPh./TiO_2_] nanofiber GCE exhibited a proper degree of capacitance with minimal resistivity and suitable stability even after 3000 cycles, which could be suitable to be utilized in SCs applications.

## Conclusion

In conclusion, the electrospinning process was used to successfully fabricate [PAN], [PAN-PPh.], and [PAN-PPh./TiO_2_] nanofibers. Because of the good synergistic effects of [PAN-PPh.] polymers and TiO_2_ nanoparticals, [PAN-PPh./TiO_2_] composite nanofibers revealed a proper specific capacitance with a minimal resistivity and outstanding cycling stability of 79.38% capacitance retention after 3000 cycles compared with PAN, and PAN-PPh. nanofiber GCEs using the charge–discharge technique. These proved that [PAN-PPh./TiO_2_] nanofiber GCE could be suitable to be utilized in supercapacitor applications. We plan on employing this simple process for synthesis to develop several types of PAN-based composites with various types of semiconductor nanostructures and metal–organic frameworks that can be utilized as supercapacitor electrodes or other energy storage devices.

### Supplementary Information

Supplementary Figures.

## Data Availability

All data generated and/or analysed during the current study are included in this published article [and its supplementary information files].
