# Supplementary material for: Electrospun network based on polyacrylonitrile-polyphenyl/titanium oxide nanofibers for high-performance supercapacitor device
Source: Sci Rep. 2024 Mar 20;14:6683. doi: 10.1038/s41598-024-56545-w (PMC10954625; doi:10.1038/s41598-024-56545-w)
Supplement: Supplementary file 1 — Supplementary Figures. [file 41598_2024_56545_MOESM1_ESM.docx]

**Electrospun Network Based on Polyacrylonitrile-Polyphenyl/Titanium Oxide Nanofibers for High Performance Supercapacitors**

**El-Refaie Kenawy^a^, Youssef I. Moharram^b^, Fatma S. Abouharga^b^ and Mona Elfiky^b^.**

**^a^** Polymer Research Group, Department of Chemistry, Faculty of Science, Tanta University, Tanta, Egypt.

**^b^** Analytical and Electrochemistry Research UNIT, Department of Chemistry, Faculty of Science, Tanta University, Tanta, Egypt.

**^*^Corresponding author e-mail:** [Elfiky_mona@science.tanta.eu.eg](mailto:Elfiky_mona@science.tanta.eu.eg&osama.abuzalat@mtc.edu.eg)

**Figure.S_1_**. TGA curve of PPh. nanofiber.

**Figure. S_2_.** XRD pattern of PPh. nanofiber.
